# Predictors of Carotid Atherosclerosis Progression: Evidence from an Ultrasonography Laboratory

**DOI:** 10.3390/brainsci12121600

**Published:** 2022-11-23

**Authors:** Stefano Caproni, Alice Riva, Giada Barresi, Danilo Costanti, Franco Costantini, Francesca Galletti, Chiara Di Schino, Elisa Appolloni, Marco Muti, Carlo Colosimo

**Affiliations:** 1 Neurology and Stroke Unit, Neuroscience Department, “S. Maria” University Hospital, 05100 Terni, Italy; 2 Neurology, Medicine Department, Università Politecnica delle Marche, 06126 Ancona, Italy; 3 University of Messina, 98124 Messina, Italy; 4 University of Rome “Sapienza”, 00155 Rome, Italy; 5 Health Physics, Oncology Department, “S. Maria” University Hospital, 05100 Terni, Italy

**Keywords:** carotid ultrasound, carotid atherosclerosis, stroke, risk factors, diabetes, large-vessel disease

## Abstract

Background and purpose: The purpose of this study was to investigate the role of risk factors in predicting the variation in carotid atherosclerosis at ultrasonographic follow-up and, therefore, its role in the progression of large-vessel disease. Methods: This retrospective population study included all the outpatients that underwent at least two carotid ultrasonographies at our laboratory from 2001 to 2017. Demographic data, vascular risk factors, and the results at follow-up were analysed to determine if correlations exist between these risk factors and variation in carotid atherosclerosis. Results: Data from 600 patients (327 males and 273 females with a mean age of 67 years) were collected. The mean follow-up period was 49 months (range: 1–195). We analysed each demographic variable and risk factor to assess its correlation with a worsening of carotid atherosclerosis; previous myocardial infarction (2.594), previous carotid surgical treatment (2.368), and hypertension (1.85) were found to have the highest odds ratios, respectively. Furthermore, the sample was divided into specific subpopulations (diabetes, hypertension, and smoking), and an association was found between age and worsening stenosis. Discussion and conclusions: Our results confirm the importance of carotid ultrasonographic follow-up in the monitoring and managing of large-vessel disease. Myocardial infarction, previous stroke, and previous surgical treatment were the strongest predictors of a worsening of carotid atherosclerosis. These findings suggest a strict follow-up is needed, even in the absence of significant carotid atherosclerosis at baseline.

## 1. Introduction

Stroke is the second leading cause of death and the third leading cause of disability worldwide; as carotid atherosclerosis (CA) is one of the main aetiologies of stroke, its prevention constitutes a pivotal objective for clinicians [1]. Additionally, CA is associated with small-vessel disease (SVD), which can cause lacunar infarcts, one of the most frequently occurring subtypes of ischemic strokes. SVD, can cause dementia, as well as other severe neuropsychiatric disorders, parkinsonian signs, and frailty in the elderly [2].

Furthermore, large- and small-vessel thrombotic events share their pathophysiological mechanisms with haematological disorders, particularly when associated with prothrombotic or thrombophylic states. Thus, the use of a comprehensive haematological laboratory test should be considered when patients suffer from CA in order to make a correct diagnosis and to establish the optimal secondary prevention strategy for recurrent vascular cerebral disease [3].

The SARS-CoV-2 pandemic outbreak in 2020 increased the worldwide burden of stroke since neurological disorders are part of the multisystem phenotype of COVID-19. A hypoxic state due to respiratory failure, diffuse thromboembolism, and inflammatory dysregulation can precipitate atherosclerotic vessel disease, leading to thrombotic stroke [4]. In addition, the increased inflammatory/oxidative state as a result of COVID-19 may cause mitochondrial malfunction, which could result in platelet damage leading to cerebral ischemic and haemorrhagic events, especially in the presence of typical vascular risk factors [5].

Therefore, given its non-invasiveness and reproducibility [6], the use of ultrasound screening is preferred over other methods in the diagnosis and follow-up of CA. The increasing demand for carotid ultrasound (CU) screening is impacting relevant public costs, creating a need for specific indicators of heart and cerebral vascular pathologies in routine CU exams [7].

Given this context, the present study aimed to investigate the predictive role of vascular risk factors in CA worsening, and to identify the patients at a high risk of developing potentially symptomatic carotid stenosis.

## 2. Methods

This retrospective study involved all of the outpatients who underwent at least two CU exams in the Ultrasound Laboratory of Neurology of S. Maria University Hospital in Terni, Italy between 2001 and 2017. CU exams were performed during routine screening or patient follow-up where vascular risk factors were present. 

The current study included patients over 18 years old. Exclusion criteria were a poor-quality acoustic window or a peculiar neck anatomy deemed unsuitable for a CU exam.

For each patient, age, gender, vascular risk factors (diabetes, hypertension, dyslipidaemia, and smoking), previous acute myocardial infarction (ST-elevation myocardial infarction) or acute ischemic stroke, previous carotid surgery, and the temporal interval between their first and last CU exams were collected. 

The degree of carotid stenosis was calculated according to standard ultrasound and hemodynamic criteria. Specifically, stenosis was considered to be at 50% if the lumen reduction (according to ultrasound evaluation) was associated with a peak systolic velocity >125 cm/s, while stenosis was considered to be at 70% if the peak systolic velocity was >220 cm/s [8]. Patients were divided into two groups, stable (S) or worsened (W), based on the absence or presence of progression in the degree of carotid stenosis at follow-up, respectively. Furthermore, this group division was repeated when defining the cut-off for worsening of carotid stenosis as either 50% (if at follow-up the stenosis exceeded the 50% value; S50 and W50) or 70% (if at follow-up the stenosis exceeded the 70% value; S70 and W70).

Demographic and clinical data were analysed using Minitab v.17 tools. Univariate and multivariate analyses were used to evaluate the predictive roles of the measured variables. Subsequently, the odds ratios for the selected variables were calculated.

The univariate analysis was conducted using a regression analysis where the independent variable was the progression of CA at follow-up, and the dependent variables were the risk factors (sex, diabetes, etc). This analysis was used to give a rough first indication of the results in terms of the predictive variables. In fact, the variance explained by the model was very low (16%) and the predictive variables with a *p*-value < 0.05 were: above-average age (*p* < 0.0001), sex (*p* < 0.0001), and myocardial infarction (*p* < 0.006).

To give a better indication of the relationships between the variables, a multivariate analysis using a principal four-component approach was conducted. Considering that some variables are dichotomous/binary and others are continuous, we used a standardised approach (correlation matrix). The results showed that a standardised eigenvalue explaining more than 15% of the variance (first eigenvalue) was not present and that 95% of the variance was explained by 90% of the eigenvalues. Furthermore, in the first four components, there was no variable with a weight greater than 40% (correlation factor). Given these results, we considered the whole set of measured variables to be predictive variables. This study was approved by the local review board of Santa Maria University Hospital.

## 3. Results

This study included 600 patients (327 males and 273 females, mean age: 67 years). The mean follow-up period was 49 months (range: 1–195 months). Demographic and clinical data are reported in Table 1. The progression of carotid stenosis was proportional to the follow-up period (25.97% of the worsening occurred during the first year, whereas 44.53% occurred during the second year, 50.48% occurred during the third year, 59.70% occurred during the fourth year, and 63.23% occurred during a period of over 4 years; Table 2).

In the first part of the study, we analysed the correlations between carotid stenosis and the demographic data (age and sex) and cardiovascular factors (diabetes, hypertension, cigarette smoking, dyslipidaemia, stroke, myocardial infarction, and endarterectomy) in the following groups:

- Stable patients vs. worsened patients (Table 3);

- Stable patients and worsened patients with a degree of worsening less than 50% vs. worsened patients with a degree of worsening more than 50% (Table 4);

**Table 4:** Analysis of the correlations between the risk factors and worsening of carotid stenosis (stable and worsened patients whose aggravation level is less than 50% vs. worsened patients whose aggravation level is more than 50%).

Risk Factors	Odds Ratio	Confidence Interval of 95%	*p*-Value
Diabetes	1.206	0.7007–2.057	0.4951
Hypertension	1.115	0.6314–1.969	0.7713
Dyslipidaemia	1.444	0.8515–2.448	0.1869
Cigarette smoking	1.630	0.8855–3.004	0.1208
Myocardial infarction	3.170	1.646–5.951	0.0010
Stroke	2.314	1.309–4.082	0.0056
Endarterectomy	5.355	2.419–11.49	0.0001
Sex	1.468	0.8592–2.462	0.1846
Above-average age	0.8224	0.4849–1.394	0.5087

- Worsened patients with a degree of worsening less than 70% vs. worsened patients with a degree of worsening more than 70% (Table 5).

**Table 5:** Analysis of the correlations between the risk factors and worsening of carotid stenosis (worsened patients whose aggravation level is less than 70% vs. worsened patients whose aggravation level is more than 70%).

Risk Factors	Odds Ratio	Confidence Interval of 95%	*p*-Value
Diabetes	1.641	0.6737–3.650	0.2754
Hypertension	0.64	0.2563–1.649	0.4348
Dyslipidaemia	0.7086	0.2813–1.672	0.5177
Cigarette smoking	1.056	0.4145–2.922	>0.9999
Myocardial infarction	3.307	1.342–8.398	0.0141
Stroke	1.986	0.7725–5.028	0.1659
Endarterectomy	17.69	6.799–45.16	<0.0001
Sex	1.260	0.5337–3.178	0.6670
Above-average age	0.7646	0.3443–1.858	0.6646

In the first group of patients, results showed that the worsening of the stenosis was strongly correlated with myocardial infarction (OR: 1.85), carotid endarterectomy (CEA) (2.368), hypertension (1.85), age (1.657), cigarette smoking (1.554), and diabetes (1.455). In the second group, the worsening of stenosis was correlated with CEA (5.355), myocardial infarction (3.307), and ischemic stroke (2.314). In the third group, we observed high ORs for CEA (5.355) and myocardial infarction (3.307).

In the second part of the study, we analysed the correlations between carotid stenosis and the demographic data (age and sex) and cardiovascular factors (diabetes, hypertension, smoke, dyslipidaemia, stroke, myocardial infarction, and CEA) in the following subpopulations:

- Diabetic patients (220) (Table 6);

**Table 6:** Analysis of the correlations between the risk factors and worsening of stenosis in diabetic patients (worsened patients: 127; stable patients: 93; and total patients: 220).

Risk Factors	Odds Ratio	Confidence Interval of 95%	*p*-Value
Hypertension	1.362	0.7307–2.517	0.3403
Dyslipidaemia	1.078	0.6335–1.829	0.7854
Cigarette smoking	1.015	0.5417–1.878	>0.9999
Myocardial infarction	1.934	0.9067–4.123	0.1306
Stroke	4.291	1.743–10.27	0.0013
Endarterectomy	1.027	0.3443–2.930	>0.9999
Sex	1.141	0.6538–1.957	0.6732
Above-average age	3.578	1.780–7.055	0.0003

- Hypertensive patients (429) (Table 7);

**Table 7:** Analysis of the correlations between the risk factors and worsening of stenosis in hypertensive patients (worsened patients: 241; stable patients: 188; and total patients: 429).

Risk Factors	Odds Ratio	Confidence Interval of 95%	*p*-Value
Diabetes	1.252	0.8409–1.842	0.2743
Dyslipidaemia	1.202	0.8227–1.760	0.3812
Cigarette smoking	1.260	0.7608–2.098	0.3822
Myocardial infarction	2.062	1.124–3.681	0.0222
Stroke	1.348	0.8173–2.245	0.3030
Endarterectomy	1.600	0.7037–3.625	0.3975
Sex	0.9199	0.6265–1.352	0.6957
Above-average age	2.716	1.704–4.347	<0.0001

- Dyslipidaemia patients (298) (Table 8);

**Table 8:** Analysis of the correlations between the risk factors and worsening of stenosis in dyslipidaemic patients (worsened patients: 165; stable patients: 133; and total patients: 298).

Risk Factors	Odds Ratio	Confidence Interval of 95%	*p*-Value
Hypertension	1.597	0.9399–2.642	0.0846
Diabetes	1.245	0.7734–1.967	0.4076
Cigarette smoking	1.494	0.8651–2.597	0.1792
Myocardial infarction	1.972	1.058–3.679	0.0347
Stroke	2.668	1.279–5.613	0.0076
Endarterectomy	1.336	0.5208–3.213	0.6508
Sex	0.8669	0.5444–1.377	0.5613
Above-average age	3.723	2.034–6.996	<0.0001

- Patients who are smokers (107) (Table 9);

**Table 9:** Analysis of the correlations between the risk factors and worsening of stenosis in smokers (worsened patients: 65; stable patients: 42; and total patients: 107).

Risk Factors	Odds Ratio	Confidence Interval of 95%	*p*-Value
Hypertension	1.002	0.4182–2.442	>0.9999
Dyslipidaemia	1.306	0.5510–2.978	0.6659
Diabetes	0.8056	0.3624–1.777	0.6921
Myocardial infarction	2.416	0.8338–6.402	0.1371
Stroke	4.074	0.8747–19.05	0.0735
Endarterectomy	1.667	0.3279–8.665	0.7019
Sex	0.6894	0.3052–1.589	0.3991
Above-average age	5.370	1.510–17.97	0.0065

- Patients with previous ischemic stroke (108) (Table 10).

**Table 10:** Analysis of the correlations between the risk factors and worsening of stenosis in patients with previous ischemic stroke (worsened patients: 59; stable patients: 49; and total patients: 108).

Risk Factors	Odds Ratio	Confidence Interval of 95%	*p*-Value
Hypertension	2.654	1.103–5.978	0.0236
Dyslipidaemia	4.094	1.736–9.275	0.0009
Cigarette smoking	5.385	1.161–25.09	0.0345
Myocardial infarction	6.641	1.528–30.50	0.0102
Diabetes	6.928	2.594–18.35	<0.0001
Sex	1.040	0.4587–2.211	>0.9999
Above-average age	3.268	1.398–7.513	0.0119

An age of older than 67 years was found to be a risk factor for all of these groups.

## 4. Discussion

### 4.1. Study of Stable and Worsened Patients

In all groups, a direct correlation was found between the duration of follow-up and the degree of worsening of stenosis (Table 2). Age was also correlated with the degree of worsening (Table 3, Table 4, Table 5, Table 6, Table 7, Table 8, Table 9 and Table 10); older age is one of the more salient risk factors for carotid atherosclerosis.

However, the role of aging in the variation in stenosis is not attributable to its action on traditional risk factors, such as lack of physical activity, cigarette smoking, hypertension, dyslipidaemia, or diabetes mellitus; it should, therefore, be considered an independent risk factor. The role of aging in the atherosclerotic process has been demonstrated by the histological studies performed by Stary and coworkers [9,10], who proved the presence of advanced atheromatic lesions in older people. The atherosclerotic process needs a wide time interval to occur, grow, and become clinically manifest. Therefore, age should be significantly associated not only with the presence of atheromatic carotid plaques but also with their extension [11] and progression [12].

Comparing the population with a stable degree of stenosis and the population in which signs of worsening of stenosis were detected (Table 3) made it possible to determine that traditional risk factors (diabetes mellitus, systemic arterial hypertension, and cigarette smoking) play a significant role in the progression of atheromatic plaque. Dyslipidaemia, despite an OR of 1.326, was not statistically significant (*p*-value = 0.0870).

Our data analysis showed a correlation between myocardial infarction (OR: 2.594; *p*-value = 0.0007) and carotid atherosclerosis. The role of carotid atherosclerosis in the genesis of heart attack is imputable to the fact that carotid plaques constitute the expression of a multisystemic pathology [13]. In addition, an increased IMT recorded using ultrasound, has been found to produce an increased relative risk of acute coronary syndromes and stroke [14,15].

Carotid plaques and complex coronary lesions are independent risk factors for the development of cardiac events; however, they can coexist as they share the same pathogenetic mechanisms [16,17].

The correlation between stroke and carotid atherosclerosis was statistically significant (Table 4, Table 6 and Table 8); this can either be a direct consequence of the rupture of the fibrous cap with local occlusion of the vessel or the outcome of a thrombotic embolism [18,19].

This study also showed that previous CEA procedures constitute risk factors for the progression of the atheromatic process (Table 3, Table 4 and Table 5). This data, although apparently contradictory, characterises the group of patients who present with a high risk of progression. This result does not indicate that CEA itself is a risk factor for carotid sclerosis progression but, rather, that the group of patients requiring an interventional procedure presents with an increased risk of developing hemodynamically significant atheromatic plaques. These findings, however, are limited by the small sample size as the total number of patients who underwent the CEA procedure was only 31.

Post-CEA progression manifested both as restenosis and as a worsening of contralateral stenosis. These conclusions are the same as the studies conducted by Raman et al. [20] and Ballotta et al. [21], who noted a progression of atheromatic plaques contralateral to the site of the intervention in patients undergoing CEA.

We subsequently analysed the stable patients and worsened patients with a degree of worsening less than 50% vs. the worsened patients with a degree of worsening more than 50% (Table 4).

From this analysis, it was possible to identify as statistically significant risk factors: myocardial infarction (OR: 3.170; *p*-value = 0.0010), stroke (OR: 2.314; *p*-value = 0.0056), and CEA (OR: 5.355; *p*-value = 0.0001). The other factors that were analysed did not reach statistical significance.

A further comparison was made between the worsened patients whose aggravation level was less than 70% and the worsened patients whose aggravation level was more than 70% (Table 5); the statistically significant risk factors were: myocardial infarction (OR: 3.307; *p*-value = 0.0141), stroke (OR: 2.314; *p*-value = 0.0056), and CEA (OR: 17.69; *p*-value ≤ 0.0001).

In terms of the pharmacological treatments usually utilised in CA patients, antithrombotic therapy at baseline was reported more often in the worsened patients in comparison with stable patients (OR: 1.7; *p*-value < 0.02). This slight significant difference, as for EAC, could be interpreted as representing an increased need for preventive treatment in patients suffering from vascular risk factors. 

Alternatively, statin therapy did not differ between the worsened and stable patients (OR: 1.2; *p*-value = 0.2). The limited sample size and follow-up duration of the study did not allow for proper consideration of the effect of statin therapy on CA progression.

### 4.2. Study of the Patient Subpopulations

In the second part of the study, we analysed the most frequent subpopulations: diabetic patients (220), hypertensive patients (429), dyslipidaemic patients (298), smoking patients (107), and patients with previous stroke (108).

#### 4.2.1. Diabetic Patients

Diabetes mellitus is a condition associated with increased risk of cardiovascular and cerebrovascular conditions, often associated with other cardiovascular risk factors.

The role of diabetes-induced hyperglycaemia in the pathogenesis of atherosclerosis can be due to local and systemic factors via inflammation, oxidative stress, and changes in the renin-angiotensin system [22]. Moreover, diabetics often have an altered lipoprotein profile, known as diabetic dyslipidaemia, in which plasma low-density lipoprotein (LDL) levels are often normal but, paradoxically, such lipoproteins, which are smaller and denser, have greater atherogenic power [23].

Moreover, CA worsening could be considered a surrogate endpoint for cardiovascular events in diabetic patients, despite the continued debate of this position among authors [24].

Data collected in the current study on the diabetic subpopulation are represented in Table 6. The analysis of this table allows us to identify as statistically significant risk factors: stroke (OR: 4.291; *p*-value = 0.0013) and age (OR: 3.578; *p*-value = 0.0003). The other factors that were analysed did not reach statistical significance.

#### 4.2.2. Hypertensive Patients

Hypertension contributes directly and independently to the development of atherosclerosis, causing changes in the *tunica media* and increasing endothelial permeability, the number of smooth muscle cells in the *tunica intima,* and the adhesion of monocytes to the endothelium [25].

Investigation into this subpopulation (Table 7) showed the following statistically significant risk factors: myocardial infarct (OR: 2.062; *p*-value = 0.0222) and age (OR: 2.716; *p*-value < 0.0001). The other factors that were analysed did not reach statistical significance.

Similar results were found in the study by Irace et al. [11], who showed an association between carotid atherosclerosis and elevated blood pressure (regardless of the presence, or not, of other factors involved in the metabolic syndrome).

#### 4.2.3. Dyslipidaemia Patients

The increase in plasmatic levels of LDLs is an independent risk factor for the development of atherosclerotic plaques and ischemic heart disease [26,27]. Hypercholesterolemia can facilitate the accumulation of lipoproteins that undergo an oxidative process [22], when in contact with endothelial cells, smooth muscle cells, and macrophages. Oxidised lipoproteins activate monocyte/macrophage chemotaxis, the first step of the atherogenic process [28,29].

Table 8 summarises the results obtained from studying the dyslipidaemia subpopulation and highlights as statistically significant risk factors: myocardial infarct (OR: 1.972; *p*-value = 0.0347), stroke (OR: 2.668; *p*-value = 0.0076), and age (OR: 3.723; *p*-value < 0.0001). The other factors that were analysed did not reach statistical significance.

#### 4.2.4. Smokers

Cigarette smoking is an independent risk factor for the development of atherosclerotic lesions in different arterial districts [25]. The smoking-related mechanisms that trigger ischemic episodes are a hypercoagulable state, increased blood viscosity, vasospasm, direct endothelial damage, mitogen action on vascular smooth muscle cells, and reduction in high-density lipoprotein cholesterol levels [25,30,31,32,33,34,35].

Analysis of the smoker subpopulation showed that age was a statistically significant risk factor (OR: 5.370; *p*-value = 0.0065). The other factors that were analysed did not reach statistical significance. The obtained results are shown in Table 9.

#### 4.2.5. Patients with Previous Ischemic Stroke

Carotid atherosclerosis can be completely asymptomatic or can manifest via stroke and transient ischemic attacks (TIAs) as a consequence of vascular occlusion [16]. According to the study by Lu et al. [25], the increase in wall thickness (measured with magnetic resonance imaging) corresponds to an increased risk of stroke/TIA.

The results of the current study in this subpopulation are shown in Table 10. This table highlights as statistically significant risk factors: hypertension (OR: 2.654; *p*-value = 0.0236), dyslipidaemia (OR: 4.094; *p*-value = 0.0009), smoking (OR: 5.385; *p*-value = 0.0345), diabetes (OR: 6.928; *p*-value ≤ 0.0001), myocardial infarction (OR: 6.641; *p*-value = 0.0102), and age (OR: 3.268; *p*-value = 0.0119). The other factors that were analysed did not reach statistical significance.

#### 4.2.6. Limitations

The present study has some limitations. First, a retrospective design was used. A prospective design may be preferred in order to reduce statistical biases and to incorporate both the evaluation of clinical follow-up and the role of potential treatment changes in affecting the CA prognosis. Second, a larger number of patients could improve the accuracy of the subgroup analyses. Finally, the evaluation of vertebral arteries, as well as intracranial haemodynamics, could significantly enhance our understanding of CA and its prognostic factors in these types of patients.

## 5. Conclusions

The present study confirms the importance of carotid ultrasound surveillance in the monitoring and management of large-vessel disease. It also confirms that “classical” cardiovascular risk factors (systemic arterial hypertension, cigarette smoking, diabetes mellitus, previous ischemic stroke, and myocardial infarction) are predictive of worsened carotid stenosis at follow-up, highlighting their key role in the pathogenesis of thrombosis. Among the risk factors, previous myocardial infarct, stroke, and previous CEA were associated with the most significant CA worsening (carotid stenosis >70% at follow-up), indicating a bidirectional link between CA and ischemic events arising from metabolic and inflammatory mechanisms. Moreover, subgroup analyses showed an important role of age in CA worsening in each of the patient subpopulations, emphasising the need for effective early treatment in patients with vascular risk factors, to reduce the risk of atherosclerosis disease progression. For patients with such diseases and risk factors, a close follow-up is needed, even in the absence of significant CA at the basal exam.

## Figures and Tables

**Table 1:** Demographic and clinical data for the patients in total and for the two groups.

Risk Factors		Total (600)	Worsened (311)	Stable (289)
Age (average)		67	65	68
Sex	Male	327	173	154
	Female	273	138	135
Diabetes		220	127	93
Hypertension		429	241	188
Dyslipidemia		298	165	133
Smoke		107	65	42
Endoarterectomy		31	22	9
Myocardial infarction		67	48	19
Stroke		108	59	49
Terapia antiaggregante		219	132	87
Terapia anticoagulante		285	156	129

**Table 2:** Evolution of the patients’ carotid stenosis across the follow-up period.

Follow-Up Period (Months)	Total Patients	Worsened Patients	Stable Patients
0–12	77	20	57
13–24	128	57	71
25–36	105	53	52
37–48	67	40	27
>49	223	141	82
0–24	205	77	128
25–48	172	93	77

**Table 3:** Analysis of the correlations between the risk factors and worsening of carotid stenosis (stable vs. worsened patients).

Risk Factors	Odds Ratio	Confidence Interval of 95%	*p*-Value
Diabetes	1.455	1.043–2.043	0.0339
Hypertension	1.85	1.284–2.653	0.0008
Dyslipidaemia	1.326	0.9617–1.833	0.0870
Cigarette smoking	1.554	1.025–2.361	0.0432
Myocardial infarction	2.594	1.485–4.479	0.0007
Stroke	1.147	0.7631–1.743	0.5258
Endarterectomy	2.368	1.062–5.310	0.0408
Sex	1.100	0.7965–1.520	0.5671
Above-average age	1.657	1.198–2.272	0.0025

## Data Availability

Not applicable.
